# Are “Extracurricular” Activities Really Extracurricular? The Activities That Matter Least in School Are the Ones That Best Teach Real-World Critical and Creative Thinking

**DOI:** 10.3390/jintelligence13010001

**Published:** 2024-12-27

**Authors:** Robert J. Sternberg, Sherry Lin, Eric C. K. Nguyen

**Affiliations:** Department of Psychology, Cornell University, Ithaca, NY 14853, USA; xl889@cornell.edu (S.L.); ecn46@cornell.edu (E.C.K.N.)

**Keywords:** curricular activities, extracurricular activities, musical training, athletic training, critical thinking, creative thinking

## Abstract

Curricula in school often do not prepare students adequately for the kinds of critical and creative thinking that they will need in their careers and lives. Part of the problem is that the characteristics of real-world problems differ greatly from the characteristics of many curricular activities, and so what the students learn in school about critical and creative thinking may fail when generalized to everyday problems. We suggest that extracurricular activities, such as in aspects of musical and athletic training, often prepare students better for real-world challenges. We describe the kinds of methods that can be used in curricular instruction to better prepare students for the challenges of the everyday world.

## 1. Introduction

When students go to school, they spend most of their time in what often are labeled as two kinds of activities: “curricular activities” and “extracurricular” activities. We define curricular activities here as those that (a) are part of the school curriculum, (b) award academic credit, (c) deliberately deliver instruction to students, and (d) are conducted during the course of regular school hours. We define extra-curricular activities as (a) outside the school curriculum, (b) typically not awarding academic credit, (c) typically not delivering direct instruction to students, and (d) typically (although not always) conducted outside regular school hours. Examples of curricular activities are English literature, mathematics, physics, biology, history, geography, and music (as part of school instruction). Examples of extracurricular activities are participating in a regional science fair, forming and playing in an informal jazz band, starting a business selling one’s cookies, participating in a debating club, Boy Scouts, All-State Orchestra, and playing on a school’s intermural football or volleyball team.

For most students, curricular activities tend to be the most important ones for their futures in terms of taking standardized tests, gaining college admissions, graduating on time, and later, obtaining employment. For example, in competitive college and university admissions, school grades and sometimes scores on standardized tests that measure knowledge and skills based on school curricula will be, for many students, the most important determinants of admission success (Lemann 1999). For some schools, curricular performance may be all that matters. The exceptions, at least in the United States, are few but significant: admissions and scholarships based on athletic or other extracurricular activities, or admissions and scholarships to specialized colleges and universities, such as musical conservatories or colleges of art (Sternberg et al. 2022). Of course, if a society decides to count test scores greatly for predicting future success (Lemann 1999; Liu 2013), the mere fact of doing well on a test may become a self-fulfilling prophecy for later success, with actual job-related skills mattering less.

An informal task analysis of both curricular and extracurricular activities, however, seems to show that it may be that certain extracurricular activities, in many instances, are often the activities that will best develop the critical- and creative-thinking skills that are vital for success and perhaps satisfaction, if not in school, then for life outside of and after the end of school (Sternberg and Spear-Swerling 1999). The reason is that the contexts of serious extracurricular activities typically are similar to those of everyday life; critical and creative thinking always occur in context, with the context greatly affecting the quality of the thinking. Before examining the basis of this conclusion, we define what we mean by critical and creative thinking.

## 2. Defining Critical and Creative Thinking

### Definitions

Although there are many definitions of both creative and critical thinking (see Kaufman and Sternberg 2019; Sternberg and Niu, forthcoming), we draw in particular on the work of Sternberg (2019) in conceptualizing critical and creative thinking. We do so, recognizing that others might use different definitions, which nevertheless we would expect to be, in general, highly overlapping with ours. Underlying both critical and creative thinking is intelligence, but in a broader sense than the term is usually used.

*Critical thinking* comprises two parts: attitudinal and cognitive. The attitudinal element concerns making an effort to think critically; the cognitive element is actually engaging in the critical thinking. The cognitive element encompasses both formal and informal inductive and deductive reasoning about information that is drawn from one’s knowledge base. Formal inductive reasoning would be, for example, what one would use to conclude that if all the many ravens one has seen are black, probably, the next raven one sees will be black (Hempel 1945). There is no guarantee that the next raven or any future raven will be black, but the inference is plausible, given what one has seen in the past. Informal inductive reasoning would be the conclusion that if one’s teacher was in a bad mood this morning, it probably would be better not to ask for a favor in the afternoon. In every case, the reasoning is cognitive, but the decision to use it, and how to use it, is attitudinal. Thinking can lead to mistaken or inadequate conclusions because people fail to decide to think critically, or because they reason erroneously. Human reasoning is prone to error, and so, therefore, is critical thinking.

Because inductive reasoning always yields uncertain conclusions, the difference between the first and second examples above is that the first is based on abundant evidence and the second on very little evidence. Informal inductive reasoning is particularly prone to errors. It is useful in everyday life much of the time but sometimes leads us astray, such as when we assume that the teacher’s mood in the morning will be representative of the teacher’s mood in the afternoon (see Kahneman and Tversky 1971).

Formal deductive reasoning is the kind of reasoning used to deal with proofs and syllogisms--for example, to decide that if all men are mortal and Socrates is a man, then Socrates is mortal (Wason and Johnson-Laird 1972). Informal deductive reasoning is the type of reasoning used to conclude that if Advertiser X made the statement that their product is the best, and we believe that Advertiser X is entirely trustworthy, then their product is the best. In this case, our informal deductive reasoning leads to a faulty conclusion, as informal deductive reasoning often does (Walton 2008). We use inductive reasoning in solving many of the humanities problems we encounter in school but utilize deductive reasoning for many of the mathematics problems we encounter in school.

Whereas critical thinking concerns the information we are given, creative thinking goes beyond the information we are given in a novel and useful or effective way (Runco and Jaeger 2012). That is, we think in a way that is new, or at the very least, new to us, and the thinking yields a result that can be applied in a useful or otherwise effective way. A theory that encompasses both critical and creative thinking is Sternberg’s (2019) theory of adaptive intelligence, as considered next.

## 3. Underlying Theory

The theory underlying this article is Sternberg’s (2019) theory of adaptive intelligence. This theory views intelligence as the set of skills and attitudes one needs to adapt to current problems, anticipate future problems of real-world environments, and create a better world, not only for oneself but also for others in one’s own generation and in future generations. In this view, both critical and creative thinking serve the purpose of adaptation to the environment. Creative thinking allows us to generate novel and potentially useful responses to the innumerable adaptive problems that face us in our lives; critical thinking helps to ensure that our responses, or other people’s, are indeed adaptive to the problems we are facing, rather than merely appearing to be adaptive at first glance.

Intelligence underlies both critical and creative thinking. As conceived of here, intelligence comprises attitudes as well as abilities (Sternberg 2022; Sternberg et al. 2024). Both critical and creative thinking involve both attitudes and abilities (Sternberg 2018).

### 3.1. Concerning Critical Thinking

Ennis (2011) suggested that dispositions in thinking, similar although not identical to attitudes, are at least as important to critical thinking as cognitive abilities. Philosophers like Richard Paul (1984a, 1984b, 1985, 1987) and Matthew Lipman (1987), and psychologists such as Diane Halpern (Dumitru and Halpern 2023; Halpern 1987; Halpern and Dunn 2021, 2023) and Jane Halonen (Halonen and Dunn 2020; Halonen and Gray 2015), have emphasized that critical thinking is a mix of abilities and attitudes. Their formulations of critical thinking involve both dispositions (related here to “attitudes”) and cognitive abilities. The taxonomy proposed by Ennis is likely the most nearly complete one available. According to the taxonomy, individuals can score well on a critical-thinking assessment but not typically think critically. The reason is that many tests of critical thinking, such as the Watson–Glaser Critical Thinking Appraisal III (Pearson, 2024), assess only the cognitive abilities of critical thinking but not the attitudes. In particular, the Watson–Glaser measures abilities involved in (1) inferences, (2) recognition of assumptions, (3) deduction, (4) interpretation, and (5) evaluation of arguments. There are no attitudinal scales.

What are examples of attitudes of critical thinking? Examples of critical thinking, in the spirit of Ennis (2011), include the following:

1. Desire to reason with validated facts rather than unproven assertions;
2. Desire to reason in a way that is logical and internally consistent;
3. Putting the importance of truth above that of feelings about what one would like to be true or what one has been told is true;
4. Skepticism of authority in the absence of validated relevant credentials of the authority;
5. Unwillingness to be swayed by emotions that lead one off a path of fact-based or logical reasoning;
6. Reflecting upon whether an argument passes a common-sense test—that is, it makes sense in terms of the way the world works;
7. Refusing to utilize, for important decisions, unvalidated shortcuts, such as satisficing (i.e., accepting one’s first satisfactory but not necessarily optimal solution as “good enough”);
8. Checking whether there are assumptions underlying one’s critical thinking that have not been shown to be valid or even tenable;
9. Ensuring that one is not putting self-interest over rational thinking;
10. Looking at all sides of an argument, not just one’s preferred side.

Consider for a moment soloists (e.g., solo pianists) and athletic training for solo events (e.g., running, singles tennis). If one wishes to become known for one’s efforts, one should have the attitude that it is important to learn what audiences will want and, ultimately, watch or even pay for (Sternberg 2020). For example, if one’s attitude is to ignore the audience and live in a “dream world”, it can be hard to make a living in anything, especially as a musician. One must think clearly about what will work with the audience and be in touch with people’s tastes. To keep people coming back, one must put together programs that are more than “good enough”, but rather that will excite audiences enough for them to want more.

Whatever one’s self-interest or preferences may be, if one cannot think critically about what audiences want, or if one is not interested in what audiences want, one will likely not thrive as a solo performer. Among vocalists, one could argue about exactly how great Taylor Swift’s musical performances are, but one could scarcely argue about her extraordinary audience appeal. This appeal is the envy of many musicians. Much the same is true of solo athletic performance: If a tennis player wants returning audiences, they must not just win but win in a way that excites the passions of the audience. Or consider diving: Quan Hongchan, a record-breaking Chinese diver, achieved a large audience not only because of her nearly perfect performance in the Tokyo Olympics of 2020 but also because of her ability to enter the water with barely a ripple.

### 3.2. Concerning Creative Thinking

Creative thinking, like critical thinking, relies heavily upon attitudes toward life and living. Creativity clearly has an ability component (Amabile 1996; Amabile and Pratt 2016; Lubart 2017; Sternberg 2018), but the more easily modifiable component is an attitudinal one. Examples of creative attitudes include the following (Sternberg 2018):

1. Defying the crowd—deciding for oneself what one thinks;
2. Defying oneself—being willing to let go of wrong or obsolete ideas or information;
3. Defying the Zeitgeist—not accepting one’s prior assumptions about the world without first examining them;
4. Being willing to critique one’s ideas and ask what possible outcomes would result from them, in particular, the best, worst, and expected outcomes;
5. Being willing to promote one’s ideas, realizing that creative ideas usually do not “sell” themselves;
6. Being willing to take sensible risks;
7. Being willing to overcome the obstacles that inevitably face creative people when their ideas frighten or antagonize other people;
8. Having the intellectual humility to recognize that no idea, even one’s own, is perfect and that there is always room for improvement;
9. Finding something one loves to do;
10. Viewing creativity as within one’s reach if one has a creative attitude toward life.

Creative thinking plays an essential role in musical and athletic activities. Being an effective tennis player, for example, is, in good part, about having a creative attitude and the skills to execute that attitude. To win, one generally must hit the ball where, and sometimes when the opponent does not expect one to hit it. If one hits in the place that the opponent expects, the opponent is more likely to return the ball. One wins in tennis, as in so many competitions, by doing what one’s opponents do not expect—what is novel and what is useful for coming out victorious. Similarly, in distinguished soloist performances of classical music, one must play the music as written but also have an interpretation that is true to the composer while also expressing one’s own creativity.

These attitudes are at least as important as any abilities because they determine, to a large extent, whether one will even try to be critical or creative in one’s thinking. Indeed, critical thinking is essential to creative thinking because when someone has a creative idea, they need critical thinking to evaluate its novelty and usefulness. There are also many attitudes that can lead one away from critical and creative thinking—that decrease the quality and quantity of one’s critical or creative output—and they can be very powerful (Sternberg 2018):

1. Believing that it is more important to go along with what others think, say, and do than to be right;
2. Believing that one’s goal in life is to do what it takes to flatter people higher up in a chain of command;
3. Being afraid to go beyond one’s past thinking;
4. Becoming entrenched in a way of thinking;
5. Accepting the assumptions that everyone else accepts because, one thinks, if everyone accepts those assumptions, they almost certainly must be right.

Why should attitudes be seen as related to intelligence, critical thinking, or any other higher-order mental construct at all? The question, to some extent, is whether one looks at test scores as predictors or as criteria. When Binet and Simon (1905, 1916) created the Binet–Simon intelligence tests, they used a rather broad range of tests, but the tests were clearly intended as predictors. The criterion was special-education placement. Should a child be placed in a special-education class for students who are unable to profit from a regular curriculum? The test score was not seen as an end in itself.

According to Sternberg (2019), both critical and creative thinking draw heavily upon executive processes of thought, or “metacomponents”, which Binet and Simon (1916) thought were central to intelligence. These processes are (a) recognizing the existence of a problem, (b) defining the nature of the problem, (c) representing the problem mentally, (d) allocating resources to the solution of the problem, (e) formulating a strategy to solve the problem, (f) monitoring the solution of the problem while problem-solving is ongoing, and (g) evaluating the solution of the problem after it is reached. Effective use of the metacomponents helps to ensure, to the extent possible, that the solution we reach is a good one in terms of utilizing effectively the information at hand and going beyond it.

### 3.3. Characteristics of Real-World Problems and Their Relation to Curricular and Extracurricular Activities

Sternberg (2019) further argued that the processes of critical and creative thinking can function in quite different ways in practical, everyday situations from the way they do in more abstract, school-like situations. That is, someone could be an effective problem solver when the material upon which one reflects is abstracted from life (e.g., numbers, symbols, or academic content outside everyday contexts) rather than practical in everyday life. Sternberg and colleagues have collected data suggesting that the two kinds of thinking—academic and practical—often yield thinking that is of different quality when compared across people (Hedlund 2020; Sternberg and Hedlund 2002). That is, different patterns of individual differences often are obtained for thinking applied to more academic versus more practical problems. This difference is crucial as a basis for the present article.

We need at the outset to clarify what we mean by “curricular” and “extracurricular.” Although the terminology implies a clearcut and perhaps even dichotomous distinction, no such clear distinction exists. That is why we put the term “extracurricular” in quotation marks in the title of this article. For example, an activity such as orchestra, band, or chorus, or such as basketball or volleyball, can be part of the curriculum, although many schools will not include such subjects in academic averages (GPAs) and schools that have class rank may exclude such subjects from being entered into how students are ranked. Moreover, some of the extracurricular activity, perhaps much of it, may be engaged in outside of the school or outside of school hours, such as in concerts or games in athletics. Similarly, preparation for a science fair may be done largely outside school but may start with in-school activities.

Extracurricular activities can include almost any activities that are done outside a school setting or outside class time. So, it is useful to delimit here, near the start, the range of extracurricular as well as curricular activities to which the arguments in this essay best apply. Many of the examples used in this essay are from music (orchestra, band, chorus) or athletics (any team at all), but many other activities would also be relevant, such as participation in a debating team, theater production, community service or other volunteer organization, dance group, peer-tutoring group, hospital-service organization, 4H and Future Farmers of America or other type of agricultural organization, school newspaper, school yearbook, school literary magazine, or Model United Nations, among others. The discussion in the essay would not apply as well to activities that are essentially extensions of the school day where one does academic activities, but after school.

For the purposes of this article, “curricular” activities are considered to meet the following:

1. Count toward any computed academic average or GPA that appears on transcripts—for example, language arts, mathematics, science, history/social studies, and foreign languages;
2. Count equally toward class rank, where relevant, with subjects like the above-named ones;
3. Are required or at least “counted” equally with the above-named subjects by selective universities for admissions;
4. Are included in international, national, statewide, and other major standardized testing programs.

The extracurricular activities to which the argument applies

1. Require systematic deliberate practice to improve performance, typically, both as an individual and in a group or team;
2. Require interaction with others in loosely structured and often unpredictable situations;
3. Require, at least some of the time, some kind of interaction with an audience outside one’s immediate peers and teachers in the school—that is, they are truly extracurricular;
4. Are recognized and valued by selective schools and colleges as worthwhile investments of students’ time. (For example, speed-hot-dog eating, although extracurricular, would not fulfill this requirement.)

Oddly, perhaps, extracurricular activities associated with schooling often offer activities and problems whose characteristics much more resemble the characteristics of real-world problems than do the activities and problems offered through the regular school curriculum and tests in those curricular activities. We use here playing in an orchestra or band or singing in a chorus as examples of musical activities, and playing on a team, such as for football, basketball, or baseball, as examples of athletic activities. Even in institutions where these musical activities are part of the curriculum, they are usually viewed as electives that are not necessarily academic in nature, and except for conservatory admission, they typically will count less, if at all, for college admissions. However, one can learn much about how to teach students from the ways in which these subjects are taught (Sternberg et al. 2022). Many other activities could be substituted instead of those that have similar properties. Table 1 summarizes the differences. It also shows how creative and critical thinking apply in real-world problems and settings.

Some theories of intellectual skills might lead us to reexamine whether extracurricular activities, such as music and athletics, should be better integrated into the regular academic curriculum. Gardner (1983, 2011), for example, includes both musical intelligence and body-kinesthetic intelligence in his theory of multiple intelligences. 

Details on differences between cognition in response to more academic versus more practical or extracurricular activities can be found in Sternberg et al. (2000), Ceci (1996), Ceci and Roazzi (1994), Nuñes (1994), Ceci and Bronfenbrenner (1985), Hedlund (2020), and Peters (1987).

First, real-world activities are typically free-response, not multiple-choice. Although one can imagine real-world activities that are multiple-choice—choosing between dance partners at a mixer or choosing between various coffee drinks at a coffee shop—even the real-world activities that are multiple-choice only vaguely resemble multiple-choice tests with four or five fixed answer options. For example, at one coffee shop, an individual might be able to choose among over 100,000 drink combinations. At a mixer, the number of possible partners may be very large, and who is available may vary by the second and quickly decrease as partners get selected by others—the options are not fixed. In the cases of the coffee shop and the mixer, moreover, there are no “right” and “wrong” answers, or at least knowable right and wrong ones. Most real-world problems, though, do not have obvious multiple-choice questions and answers at all. One must construct answers. In an orchestra, the notes may be given, but issues such as the exact pitch at which to play the notes, the tempo, the use or nonuse of vibrato, and the pressure on the bow of a violin—all are up for grabs. And they will be dictated in part, but not fully, by an orchestra conductor. On a football team, the situation changes by the second, and whether to go for a ball, tackle an opponent, play defensively, or just run can change in a flash. A coach can give general strategies but cannot tell players moment-to-moment what to do. There are no fixed multiple-choice options that just remain the same as one considers the problem in its dynamic nature.

Second, in real-world problems, there typically is no uniquely correct answer. Indeed, typically, there is no identifiable correct answer at all, at least at the time. How one plays notes or where a football player should move on the field are problems where the answers are indeterminate, and there is no way even of being assured that a given choice among the often-innumerable possibilities is the best. Rather, one is in a situation where one uses one’s experience and reasoning powers to choose what seems to be an optimal answer at the time or an answer that is as close to optimal as one can get. A typical real-world problem is deciding how to allocate time and resources in one’s day. For sure, there is no clear way of saying the optimal length of each activity or even what activities should be done at all besides those that absolutely must be done. The athletic and musical activities are very much like those in real life; the activities in curricular instruction generally are not.

Third, real-world problems are ill-defined. They are not clearly spelled out for the problem solver in the way that test problems typically are or in the way that problems typically are on homework assignments given by teachers. In an orchestra, for example, if a note sounds less than optimal, a failure of intonation will be noted, but there often is no one there saying that the fault is with the bow pressure or bow speed or finger pressure or color with which one tries to play the note. In a basketball game, no one would even have time to spell out during active play what a problem is and how to solve it. There simply is no time because the game evolves quickly and dynamically. Only when there are time-outs does one get a chance to consider how the team’s overall strategy—not just the strategy of the individual—should change.

Similarly, in a real-world problem, such as making a meal from scratch, if the meal does not taste the way it is supposed to, it is often hard to identify exactly what went wrong at what stage of the process. One may have to taste the meal carefully and then decide what possible causes of the miss could be, such as over- or under-cooking, use of a wrong ingredient, use of too much or too little of an ingredient, improper timing of adding ingredients, and so forth.

Fourth, real-world problems are ill-structured. There is no clearly spelled out way to get from beginning to end, and sometimes, a path that has worked before does not work again, such as when one courts a romantic partner and discovers that what worked the last time with the last partner does not work this time, or that the way of persuading one’s last boss does not work with a new boss. One then must find a new strategy, perhaps changing the strategy as one goes along and gets cues as to what is working and what is not. Orchestra members often find that the programming or way of playing that suits one audience particularly well suits a different audience only poorly. The musical director may decide that a different approach is needed with a different audience. Certainly, any academic who has spoken to varied audiences finds that the strategy that worked with a certain kind of audience in a certain place and time does not work with a different audience at a different place and time.

Seasoned scholars, for example, often adjust their talks as to whether audiences are academics or practitioners. Basketball teams research their opposition, realizing that each opposing team has different strengths and weaknesses and that the strategy that may have worked in a previous game will not work, for example, if a new opposing team is stronger on defense but weaker on offense than the last team, or if there is one star of the team who keeps getting the ball.

Fifth, real-world problems are unstable. The nature of the problem often changes from one moment to the next. For example, in athletic competitions, a team can seem at one moment to be winning and in an offensive position, only quickly to discover that their position has changed. In music, a piece can be going well so that it seems that the orchestra merely needs to get through it as planned, and then, somehow, things go off—the musicians get out of sync with each other, one section underperforms, a string player’s string breaks, the audio equipment malfunctions, or whatever, and one finds oneself in salvage mode—how can the piece be salvaged with the least damage? Many of us who have done auditions have found that an audition piece can be going well, and then we forget the music and find ourselves struggling even to finish the piece. In wars, very few things are predictable, and the nature of the war itself can change very quickly because of unexpectedly weak performance by one’s own military or unexpectedly strong performance by the opposing military.

Sixth, significant real-world problems are generally complex. When to have children, when to get married, when and where to move, what job to take—these complex problems defy the nature of academic and especially standardized test problems, which usually take a matter of seconds or, at most, a few minutes to solve. If one were to try to solve significant real-world problems, such as job choice, in the few seconds or minutes allotted for ability and achievement test problems, one almost certainly would do a very bad job solving them. One needs a different time and investigative perspective when solving most significant real-world problems from what one needs in taking a test. Even real-world problems that need to be solved quickly are complex. For example, when an orchestra piece or an athletic competition starts to go badly, it is often for reasons of interaction among the team members. Finding what went wrong with the interaction involves considering many possibilities, more than typically would be the case of the possibilities presented by problems on standardized tests.

Seventh, significant real-world problems are emotionally laden. Auditions for joining musical groups or athletic teams, for instance, are often very emotional. As a real-life example, the 2024 U.S. presidential election generated significant amounts of emotion, not only in the United States but around the world. Rarely, if ever, in most people’s lifetimes did the stakes seem so high. Although there was some impressive logical reasoning, many people found themselves going off the deep end. They could hardly think about the election without becoming highly emotional. Each side seemed, at some times and in some places, to believe that the other was intent on destroying the country.

When one is as overwrought as many people are when they are under great stress, it becomes difficult to think clearly or even with any degree of rationality. The result is problem-solving that looks little like that required by standardized tests of intelligence and achievement.

Eighth, the stakes are high in many real-world problems. In the United States, the so-called “World Series” is the most significant baseball event of the year. The pressure to perform is intense. Similarly, a major concert can put a conductor and performers on edge, resulting in their solving problems in ways they might not if the stakes were not so high. They may be more cautious, feeling they cannot afford to mishandle an event of great significance. However, the result may be impaired problem-solving or a kind of anxiety that makes it hard to think clearly and to come to a conclusion that is validly evidence-based.

Of course, there are many real-world problems of little significance, say, the choice of a breakfast cereal or whether to wash one’s hair at the beginning or end of a shower. But when the stakes are high, people realize that making a mistake can be costly—when investing money, allocating significant amounts of time to a task, or deciding whether to have children. People simply cannot solve problems of a high order with simple reasoning. They must take into account the consequences of making a mistake and then find a strategy that can maximize their potential gains while, hopefully, minimizing their potential losses. Moreover, for important decisions, other people’s futures may also be at stake, especially if one has a family or others who rely upon one. Often, arriving at a solution involves taking multiple and diverse needs into account.

Ninth, most real-world problems of consequence are solved in groups. Serious world problems, as solved by national governments or, perhaps, at the United Nations, always need some kind of consensus. In academic administration, there are few serious problems one can solve without getting some kind of consensus from at least one group, and usually, more than one group. What makes administration so difficult is that different stakeholders almost inevitably have very different ideas about what the right thing to do is. But without at least some consensus, especially among powerful constituencies, nothing can get done. And that is how real-world problem-solving is—extremely difficult to get done without the consensus of important and often powerful stakeholders. In musical and athletic activities, many tasks also require extensive cooperation, like choral/orchestral performance, collaborative songwriting, and athletic competitions. In contrast, many school-based academic problems, even “group projects”, do not explicitly teach students how to collaborate effectively (Sternberg et al. 2022).

Tenth and finally, real-world problem-solving is value driven. It is often hard to achieve consensus simply because different values lead stakeholders to different decisions about what is valuable and what is not in a solution. In athletics, some individuals and teams seem to value winning at all costs and are willing to cut corners. Others value more a sense of fair play. In music, some musicians will change their interpretation of a piece because they value a positive audience reaction the most. Others value the composer’s interpretation more.

Consider, for example, STEM disciplines. A typical course involves memorizing theories and findings and solving presented problems of one kind or another. Even lab courses typically involve doing canned or other pre-prepared laboratory exercises. But much of actual scientific endeavor is in problem finding—figuring out what problems are worth solving that can be empirically addressed (e.g., Sternberg 2016; Zuckerman 1983). Problem finding is more likely to occur in independent research that is not a part of a typical course. An example would be research that is prepared for presentation at a science fair, Future Problem-Solving endeavor, or in some other context that goes way beyond the classroom.

Future Problem Solving (https://fpspi.org) is a good example of how an activity that goes beyond the classroom can develop critical and creative thinking skills. The participants reflect upon current global issues and where they will end up in the future, considering possible solutions and obstacles to those solutions. The program encompasses community projects, creative writing, storytelling, and more. It uses a six-step approach to problem-solving that encourages both critical and creative thinking: (1) identifying challenges, (2) selecting an underlying problem, (3) producing solution ideas, (4) selecting criteria, (5) applying criteria to top solutions, and (6) developing an action plan.

There is empirical evidence that whatever academic schooling may do to raise IQ (Ceci 1996), it does not seem to do much to support critical thinking (Stanovich 2009, 2021; Stanovich and West 2000; Stanovich et al. 2013) or creative thinking (Robinson and Aronica 2015). There is evidence, however, to support the argument that extracurricular activities enhance critical and as well as creative thinking (Cotter et al. 2016; Harvey and Seeley 1984; Kuhar and Sablijic 2016).

## 4. Threats to Critical and Creative Thinking

Critical and creative thinking in the real world do not occur in a sterile environment. Rather, they occur in a world in which environmental forces can impede the quality of thought, sometimes drastically. There are various threats in the world today to both critical and creative thinking. Some of these threats are summarized in Table 2. Consider just a few.

Critical and creative thinking in the real world must endure threats that are scarcely relevant, or not relevant at all, to thinking of an academic nature. Some of these issues were discussed above as factors in real-world problem-solving. Here, they are discussed as threats. What are some of these threats?

1. **Emotionally arousing situation.** A situation can be emotionally arousing in a positive way, such as excitement about a romance, or in a negative way, such as learning that one is in danger of some kind. School curricular problems are rarely emotionally arousing. They typically do not even incite any emotion at all beyond feelings, in some cases, of boredom. But auditioning for a place in an orchestra, band, or chorus, or playing a high-stakes concert where critics, amateur or professional, are ready to pounce, is likely to be an emotionally arousing experience. Playing in a high-stakes athletic competition also can be extremely emotionally arousing and even nerve-wracking. An advantage of musical and athletic training is that one encounters these kinds of situations on a regular basis and hopefully learns to handle them the best one can. How real-world activities are executed will depend in large part on the particular motivation of the individual to use their practical skills (Dweck et al. 2004) in periods of emotional arousal.
2. **High-stakes situation**. In high-stakes situations, where our life, the life of our family, or simply our or our family’s well-being seems to depend upon a certain outcome, we may decide that critical thinking is nice, but it is our life at stake. Sometimes, such a decision is made because we have not practiced critical and creative thinking in high-stakes situations, so we respond automatically (Kahneman 2011). Often, such responses are not optimal but rather satisficing responses, but when under the stress of a high-stakes situation, we may fall back on our automatic repertoire. Having experience in athletics, music, or other activities where one has major events, such as a final audition or a final race, can prepare one for future high-stakes situations. Dealing largely or even only with low-stakes situations, however, does not give one this kind of preparation. Most school situations are of low stakes. Some are for relatively high stakes, such as standardized testing, but they typically are encountered so rarely that one may not be learning how to handle the situations.
3. **Situation with a perceived severe penalty for making a mistake**. In a situation where there is a severe penalty for making a mistake, one may be reluctant to act in any way that poses any risk at all. And with internet mobs and trolls rampant, even a small mistake can be costly.
4. **Believing false information**. Much of critical thinking today is in the process of distinguishing the enormous amounts of false information from the kernels of true information that make their way onto the internet and social media. One of the advantages of extracurricular training, such as in music and athletics, which involves high stakes, is that through repeated exposure to games or concerts, false information tends to drop out. Whatever one may have been told about a competitor or about an audience, when one meets up with them on multiple occasions, one learns that initial information may be wrong and that one must figure out for oneself what is true and what is not. No matter what one may have been told about a competitor’s backhand, for example, in a meet, they either use their backhand to advantage or they do not. In academic preparation, one spends most of one’s time preparing, so one may never get to see what the situations are like that one must actually confront in one’s life.
5. **Fear of unanticipated threats**. In current times, people live in fear of unanticipated threats. Cynical politicians and leaders magnify these threats and then present themselves as the only ones who can remove the sources of fear. In anxious times, such pseudo-transformational leadership often convinces people. The public wants someone who will take the fear away. Critical and creative thinking can help people realize that cheap appeals to fear and the hollow promise of removing fear are worth very little.

## 5. What Is to Be Done?

The solution to the problem of curricular activities often not encouraging real-world critical and creative thinking is not to do away with curricular activities, obviously, but rather to make these curricular activities more like extracurricular activities so that the curricular activities teach students how to deal with the problems of the everyday world.

There are many ways that students can learn curricular material so that they will be better prepared to think critically and creatively when they leave school. Almost any of these ways is within the reach of any school. We describe each kind of learning only briefly here. Our goal is merely to show the wide range of options that exist to make curricular learning more resemble the positive features that are shown by learning through extracurricular activities. The modes of practical learning for critical and creative thinking discussed here are summarized in Table 3.

Although the modes of practical learning are defined and exemplified in Table 3, they are not further classified. The reason is that, in practice, they each can take such different forms and thus overlap in varying amounts, depending on how they are executed. They should be looked at, therefore, not as discrete and separable but rather as overlapping ways for schools to achieve the same goal of preparing students to learn and perform in ecologically complex and challenging environments that will resemble the environments that they someday will live and work in. The terms used are ones that are used in schools, so they are listed at length to cover the various terminologies used in schools, although in somewhat different ways in different schools. These strategies, among others, follow from some of the techniques used in Grigorenko et al. (2002), Sternberg et al. (1998), and Sternberg et al. (2014).

### 5.1. Practical Learning

Practical learning involves learning that focuses on the practical applications of what is learned in school (Sternberg and Spear-Swerling 1999). For example, one might be asked how to apply knowledge of statistics to infer whether a particular drug is more effective than nothing at all for an illness one has. Then, one might ask whether the effect is both statistically *and* practically significant to matter at an individual level. The other examples below are special cases of practical learning.

### 5.2. Internships

An internship is a learning experience that involves students working in a professional setting of some kind that is supervised and structured. The student acquires work experience in their field of study while, ideally, relating what they learn to the academic work that prepared them for the internship.

### 5.3. On-the-Job Training

On-the-job training is learning done by new employees, but also, much of the time, more experienced employees who acquire, through the job, both the explicit and implicit (tacit) knowledge they need to succeed in the job (Sternberg and Hedlund 2002). Often, the on-the-job training is accomplished by doing one’s job, but it also may involve explicit training programs administered by employers.

### 5.4. Work-Study Training

Work-study training typically is a program for high school or college students that provides part-time jobs that provide financial benefits as well as practical work experience.

### 5.5. Service Learning

Service learning combines service to the community with learning that applies what students have learned in the classroom to their service to help with the real-world needs of those they serve.

### 5.6. Project-Based Learning

Project-based learning places students in a position where they develop projects that address real-world issues. Working on the projects helps develop both critical- and creative-thinking skills. Such projects may be carried out individually or in small groups.

### 5.7. Practicums

Practicums are usually highly structured learning experiences in which students can apply the knowledge they have learned through the curriculum to real-world settings. Some fields require practicums for licensing or other credentials to practice in the fields.

### 5.8. Action Research

Action research is research that studies a real-world problem while, at the same time, helping to create a practical solution to that problem. The research is done in the real world, not in a laboratory or a classroom, and is designed to effect change. The research often lacks the rigor of laboratory-based research.

### 5.9. Field-Based Learning

Field-based learning takes place in real-world settings rather than in a classroom or laboratory. Students interact directly with the world and learn from those interactions. Thus, such learning occurs through experience in the world, not through textbooks or labs.

### 5.10. Experiential Learning

Experiential learning is learning by doing. Whatever one wants to learn about, one learns by doing it rather than by reading about it or reading about others doing it.

### 5.11. Problem-Based Learning

Problem-based learning is learning by solving problems, usually those that are currently being addressed in the everyday world. The students are given problems and are asked to solve them. By solving those problems, the students can learn the skills that they might have learned in a decontextualized way through expository learning.

### 5.12. Simulation Learning

Simulation involves students operating in environments that are like the real thing—whatever they are setting out to learn—but that are controlled and safe. If the students make a mistake, there are no real-world consequences because what they were doing was a facsimile of the real thing rather than the real thing itself. For some kinds of jobs, such as commercial airplane pilots, simulations are a necessary way of learning.

### 5.13. Vocational Learning

Vocational learning prepares students for particular occupations, often trades. The term is often applied to labor that does not require advanced education, such as that obtained in college, for learning to do a certain kind of work, such as plumbing, carpentry, and crafts of various kinds.

### 5.14. Study Abroad

Study abroad is an organized educational program of education that is carried out in a country other than the one the student lives in. Often, the goal is not only to study the typical curriculum for one’s level of education but also to learn about the culture of the country in which one studies. If the country uses a language with which one is not familiar, one also may decide to learn the language to some level of mastery. Study abroad, unfortunately, often is limited only to those whose families can afford the costs associated with it.

### 5.15. Performance-Based Learning

Performance-based learning involves the student demonstrating their learning through a performance of some kind, which could be an exhibition, a portfolio, a project, or some other active demonstration of the real-world application of what one has learned.

### 5.16. Lifelong Learning

Lifelong learning is simply learning that does not stop when one finishes formal schooling, but that continues, in whatever form, throughout the duration of one’s life.

### 5.17. Community Need-Finding

A team of students identifies a problem or need in their community and, with the collaboration of relevant resources in the community, works to help solve or at least alleviate the ill effects of the problem.

### 5.18. Career-Technical Learning

Career/technical education (CTE) is generally used in disciplines such as agriculture, various trades, business and marketing, consumer sciences, health sciences, and especially technology education.

### 5.19. Capstone Courses

Capstone courses typically provide an integrative experience at the end of an academic career at a particular level, such as in the senior year of high school or college. They are supposed to provide an opportunity to reflect upon and synthesize what one has learned. They usually have a performance, portfolio, paper, exhibition, or some other kind of product in addition to or instead of an examination.

## 6. Implications for Education

The major implication of this article is that schools, to prepare students for real-world critical and creative thinking, should make more of their learning resemble extracurricular activities that teach real-world skills that are often absent from curricular learning. These skills include performance in real-world action, cooperation, teamwork, learning and working under stress, learning and working for high stakes, having high standards for performance, interpersonal relations, and intrapersonal relations—a better understanding of oneself. Almost any course can be taught in a way that draws upon the kinds of practical learning described above. Such teaching would better prepare students for the challenges of critical and creative thinking they will face in the everyday world, not just in the classroom.

Here are three immediate implications for educational policy:

1. *Do not assume that students will learn to think critically; embed teaching for critical thinking into existing curriculum.* Too often, educators believe that their curriculum somehow automatically teaches critical thinking. It may, but too often, it does not. All academic subjects can be taught through problem-based learning that embeds instruction in the metacomponents described above, such as recognizing the existence of a problem, defining the problem, and setting up a strategy to solve the problem.
2. *Do not assume that students will learn to think creatively through normal instruction. And do not allow teaching to the test to squelch teaching for creative thinking.* Students should be taught to deploy creative attitudes, such as redefining problems, taking sensible risks, and persuading others of the value of their creative ideas.
3. *Create an atmosphere that encourages, recognizes, and rewards both critical and creative thinking.* Too often, students are rewarded primarily or exclusively for the acquisition of declarative knowledge. Acknowledge and reward students for their critical and creative utilization of that knowledge.

There are practical barriers to implementing the kinds of reforms this article suggests. First, teachers do not always know how to teach critical and creative thinking, and schools may not have the money to teach them. Second, most schools, at least public ones, are locked into statewide or nationwide testing, and their priorities may be set by the tests. Third, reward systems in schools do not always particularly value critical and especially creative thinking. To make the necessary changes, the schools may need to change not only how they teach but what they reward.

Some countries recognize that traditional academic education often does not provide the everyday skills students need, especially those not planning to go on to college. These countries provide options that prepare students for employment in a way that is constructive (rather than the “vocational education” that is often viewed pejoratively in the United States). For example, most of Germany has the option of a Real Schule, which covers grades 5 through 10 and is designed to create a seamless transition into the workforce by having students gain experience in the kind of work they plan to do. The German Hauptschule is even more oriented toward the workforce and typically ends in grade 9. It is the least academic option. (The most academic option is the Gymnasium, which goes to grade 12 or 13). German students thus have three options to prepare for their future careers, with a choice that enables them to match their skills and interests to the kind of secondary school they attend.

There is one last caveat that must be mentioned. Critical and creative thinking must be taught in a way that helps to create a better world—that can make a positive, meaningful, and potentially enduring difference to the world (Sternberg 2019). Too much of critical and creative thinking is being used darkly today (Cropley and Cropley 2019; Cropley et al. 2008, 2010; James et al. 1999; James and Taylor 2010; Kapoor and Kaufman 2022a, 2022b, 2022c; Sternberg 2024). We need to emphasize that our responsibility to the world is to make it even a little bit better for our having lived in it, not to make it worse. Everyone can make at least some positive contribution if they just try to.

The key to success in musical activities such as orchestra, band, and chorus, and in athletic activities such as football, basketball, and tennis, is that success depends upon *ecologically valid performance*, which is performance that is relevant to real-world pursuits in naturalistic environmental contexts. What one does in extracurricular activities of those kinds is what one does when one does the work for real—outside even the remote context of the school. Students in extracurricular activities learn how to perform in the real world. In contrast, curricular performance is real performance, but it is not ecologically valid. For the most part, what one does as a student of science is not what one does as a scientist, except at advanced levels of training. And that is why extracurricular learning is so important—it teaches one to think critically and creatively in the actual circumstances that, someday, perhaps soon, one will need to think in one’s life.

## Figures and Tables

**Table 1:** Comparison of Real-World Problems to Curricular and Extracurricular Problems.

Characteristic	Real-World Problems	Orchestra/Band/ Chorus Training	Team-Based Athletic Training	School-Based Academic Problems	Roles of Creative/Critical Thinking
**Free response–Not multiple-choice**	Always	Always	Always	Sometimes	Creating a response and analyzing its quality
**Lacking a uniquely correct answer**	Usually	Usually	Usually	Sometimes	Creating a response and deciding it is good enough, although not perfect
**Ill-defined: The exact problem is not specified or often even pointed out to exist**	Usually	Usually	Usually	Rarely	Creatively figuring out what the problem is and then analyzing whether that is indeed the problem
**Ill-structured: Not clear how to get from beginning to end**	Usually	Usually	Usually	Sometimes	Creating a path to problem solution and then analyzing whether it is a useful path
**Unstable—the nature of the problem changes as one solves it**	Usually	Usually	Usually	Rarely	Creatively coping with the continuing novelty of the changing problem and ensuring one is correctly tracking the changes in the problem
**Highly complex**	Usually	Usually	Usually	Rarely	Requiring high, not trivial levels of creativity and analysis to ensure that the idea is novel and useful
**Emotionally laden**	Sometimes	Sometimes	Sometimes	Rarely	Requiring critical thinking to ensure that the creative idea is not off-track as a result of emotional reaction
**For high stakes**	Sometimes	Sometimes	Sometimes	Sometimes	Requiring creative and critical thinking for problems that matter, not for trivial test-like ones
**Team-based: Solved in groups**	Usually	Usually	Usually	Rarely	Requiring one to create and critique as part of a team, taking into account others’ ideas
**Consequential for many people**	Often	Often	Often	Rarely	The creative and critical thinking are for problems that matter and have real consequences
**Multi-stage, in that the solution to the problem becomes the next problem**	Often	Often	Often	Rarely	The creative and critical thinking occur over time and problem stages, rather than being applied to simplistic problems that can be quickly solved
**Messy**	Often	Often	Often	Rarely	

**Table 2:** Threats to Critical and Creative Thinking.

Type of Threat	Definition	Example	Problem
**Emotionally Arousing Situation**	An intensified emotional state caused by one’s environment/situation	Learning that your ex- will be watching your solo violin performance, causing you to not focus on actually playing the piece	One focuses on one’s emotional response and the emotions it arouses rather than thinking critically and creatively about the task at hand
**High-Stakes Situation**	A situation in which the well-being of oneself or a member of one’s inner circle depends on the outcome of the situation	A perceived once-in- a-lifetime audition to a philharmonic orchestra, causing one to pick an easier piece to play rather than a more challenging one	The high-stakes situation can cause a fight-or-flight response, leading one to fall back on simple/automatic thinking rather than on critical and creative thinking
**Situation with a Perceived Penalty for Mistakes**	A risky situation that can lead to a negative outcome	Your conductor threatens to throw a cymbal at your head if you play out of tune, causing you to perform with robotic precision rather than emotion	If someone perceives the risk of a situation as being too high, it may hinder their ability to think critically and creatively, as they might become too focused on potential negative outcomes
**Believing False Information**	One not being willing or able to distinguish between false and true information	You read an article saying that a new energy drink will enhance performance, so you take it before a concert, to your own detriment	By believing false information, future decisions that require critical and creative thinking may be based on a false foundation of inaccuracies
**Fear of Unanticipated Threats**	Anxiety over potential, unanticipated disruptions	You are so anxious over something going wrong during your performance (a string breaking, someone coughing randomly, etc.) that you are not focusing on your interpretation of the piece	If one’s mind is preoccupied by future threats, then the preoccupation may result in short-sighted thinking rather than critical and creative thinking

**Table 3:** Strategies for Teaching Critical and Creative Thinking in Practice.

Type of Strategy	Definition	Example
Practical Learning	Using information learned in real- world scenarios	Applying physics principles to lift a weight in the easiest way possible.
Internships	Learning in a structured, supervised, and professional setting	Learning how to prepare a stock pitch at an investment firm
On-the-Job Training	New employees acquiring knowledge while working	A new line cook first learns how to use the frying station and then, when mastery is achieved, is rotated around the kitchen
Work-Study Training	Part-time jobs given to those typically in school	A high school student is allowed to work at their school’s library
Service Learning	Community service is done with emphasis on using practical knowledge learned from class	Skills learned in a cooking class are applied to working at a soup kitchen
Project-Based Learning	Learning that involves applying knowledge to a project that will address real-world issues	Engineering students are given the task to come up with a way to reduce greenhouse gas emissions from a city
Practicums	A short-term, highly structured learning experience typically built into a student’s curriculum	A student in nursing school takes the vital signs of patients while the student is under supervision
Action Research	Research that studies real-world problems while attempting to create a practical solution to the problems	A student observes whether adding a roundabout to an intersection will decrease traffic congestion
Field-Based Learning	Learning that takes place in the real-world instead of classrooms or labs	Ethology students go into the jungle to study gorilla behavior instead of just reading about the behavior in a textbook
Experiential Learning	Learning by performing a task rather than reading about it	A student learns to throw a baseball by practicing different throwing techniques instead of just learning the biomechanics of throwing in a textbook
Problem-Based Learning	Learning by solving a given everyday world problem	Students are given the problem of reducing homelessness in a city and are asked to provide a 5-step plan
Simulation Learning	Learning in an artificial environment in which there are no real-world consequences for making a mistake	A pilot in training practices landing an airplane on a computer simulation
Vocational Learning	A curriculum made to teach someone a particular occupation/trade	A student acquires first-hand practical knowledge about welding at a trade school
Study Abroad	Education that is carried out in a foreign country (typically, in part, to learn a new language)	A U.S. born student spends a semester in France to learn French and about French culture
Performance-Based Learning	Students demonstrate their knowledge through a portfolio, project, etc.	An art student creates a portfolio with a certain theme, to be assessed at the end of the semester
Lifelong Learning	Learning that does not stop after formal education	A doctor keeps up with the current medical research to best treat their patient
Community Need-Finding	Doing observations in a community, finding out about their needs, and helping to meet these needs	A student observes that too many people are throwing their trash out their car windows as their cars move, and starts a PR campaign to discourage such behavior
Career-Technical Learning	Career technical learning is a type of curriculum that integrates academic and practical knowledge	A course where an EMT learns how the body functions in an academic setting and then how to treat emergencies in a practical setting
Capstone Courses	An integrative experience toward the end of one’s education	An undergraduate student writes an honors thesis to demonstrate the knowledge and skills they have gained over their undergraduate career
